# Haematologists’ experiences implementing patient reported outcome measures (PROMs) in an outpatient clinic: a qualitative study for applied practice

**DOI:** 10.1186/s41687-019-0166-6

**Published:** 2019-12-28

**Authors:** Stine Thestrup Hansen, Mette Kjerholt, Sarah Friis Christensen, Bibi Hølge-Hazelton, John Brodersen

**Affiliations:** 1 Department of Haematology, Zealand University Hospital, Vestermarksvej 9, 1.sal, 4000 Roskilde, Denmark; 2 Department of Regional Health Research, Faculty of Health Sciences, University of Southern Denmark, Odense, Denmark; 3 Faculty of Health and Medical Sciences, University of Copenhagen, Copenhagen, Denmark; 4 The Research Support Unit, Zealand University Hospital, Roskilde, Denmark; 5 Department of General Practice, Institute of Public Health, Faculty of Health Sciences, University of Copenhagen, Copenhagen, Denmark; 6 Centre of Research & Education in General Practice, Primary Health Care Research Unit, Copenhagen, Region Zealand Denmark

**Keywords:** Qualitative study, Haematology, Patient reported outcomes, EORTC QLQ-C30, OEQ, Applied research

## Abstract

**Background:**

The patient-doctor relationship is crucial to provide person-centred care, allowing the alleviation of symptom burden caused by disease or treatment. Implementing Patient Reported Outcome Measures (PROMs) is suggested to inform the decision-making process and lead to initiation of care. Yet there are knowledge gaps regarding how meaningful it is to incorporate PROMs in clinical settings. The aim of this study was to investigate haematologists’ experiences when PROMs were implemented in an outpatient setting.

**Methods:**

Fourteen participant observations, 13 individual interviews and three in-depth interviews were conducted with haematologists, guided by the qualitative methodology Interpretive Description. Analysis was inspired by Habermas’ critical theoretical framework.

**Results:**

The haematologists included were characterised by dichotomous experiences with PROMs, either resistant to or supporting their implementation.

None were observed to elaborate on PROMs during consultations: instead, primary attention was spent discussing the hematological agenda dictated by the system.

**Conclusion:**

The use of PROMs for individualized care was linked with extensive uncertainties and PROMs were not requested by the haematologists. To improve individualized care, other approaches may be more suitable. If PROMs are to be incorporated into future clinical practice, they should be tested tothe specific patient group and involve relevant users.

## Background

Efforts have been made by the European Hematology Association Scientific Working Group and the American Society of Hematology to develop a conceptual framework for Patient Reported Outcome Measures (PROMs) integration in clinical haematology care [1, 2]. The framework builds on the assumption that the patient-doctor relationship is crucial in providing holistic, patient-centred care and alleviating the symptom burden caused by the disease or treatment. PROMs can help identify symptom burden and inform the decision-making process, leading to initiation of supportive care [1–3].

Internationally, PROMs are recognized as a means for patients to provide information about their quality of life, symptoms, and experiences of care [4–6]. PROM assessments have the potential to introduce the patient’s perspective into clinical processes [7, 8] via self-report instruments completed by the patient but chosen by the institution [2, 9, 10]. This approach is attracting growing attention by patient organisations and by public health authorities seeking to promote and standardize the use of PROMs in clinical healthcare settings as new health technology aims to provide person-centred care at a lower cost [11–14]. Hence integration of PROMs in clinical practice has been described as the next step, as previous studies have found that the use of PROMs in routine medical care is associated with improved patient-physician communication, enhanced shared decision-making, improved symptom management, and greater satisfaction with care, as well as improved overall quality of life [4, 5, 15]. However, a critical appraisal of PROMs is lacking: adaption of PROMs from clinical research into clinical practice has potential pitfalls, as PROM data used to describe groups in research should differ from PROMs used to reflect patients’ individual health [16]. Adaption of PROMs has also been linked to measurement uncertainties, including content validity (content relevance and content coverage) and the psychometric properties of the PROM(s) [17, 18]; inadequate measurement properties could potentially lead to clinicians using invalid outcomes when consulting with patients, which again could potentially be harmful and detrimental to the patient-doctor relationship. Important knowledge gaps remain regarding the complexity of PROMs usage and how to adapt them across different settings for routine clinical care [3, 4]. Despite an extensive volume of literature on the use of PROMs for routine clinical care, it is difficult to reach firm conclusions due to the broad variety of interventions within setting-specific studies [4, 19, 20]. Research is needed on the actual experiences of users of these tools [11, 15, 21, 22]. Therefore, the aim of this study was to investigate haematologists’ experiences when implementing PROMs in clinical practice in an outpatient setting, as part of a multimethod intervention study. For an overview of the multimethod study, visit Additional file 1.

## Methods

### Study design

This study was guided by the qualitative methodology *Interpretive Description* (ID), including a focused ethnographic approach [23], to fit the nature of the aim [24]. Use of this methodology was driven by the rationale and logic inherent to applied practice, permitting the researcher to apply and combine methods as needed during the research process to fully answer the research question and identify implications for practice [24]. *Focused Ethnography*, with participant observations, interviews, and in-depth interviews, was applied to enhance the setting-specific, problem-focused and short-duration consultations between haematologists and patients [25]. Finally, the theoretical framework on critical theory by Jürgen Habermas inspired the interpretation and discussion of the data.

The PROMs applied were the European Organization for Research and Treatment of Cancer Quality of Life Questionnaire Version 3.0 (EORTC QLQ-C30) [26] and the Outcomes and Experiences Questionnaire (OEQ) [27]. For additional information on the PROMs implemented, please visit Additional file 2. Completed EORTC QLQ-C30s were transferred into the electronic medical record system using a standardized short-form [28] while OEQs were transferred in full. A detailed description of the completion process is described by Hansen et al. [29].

### Participants and setting

Thirteen haematologists performing consultations in a haematological outpatient clinic were included, sampled with maximum variation to reflect departmental variation in gender, age, educational background, experience, and ethnicity, striving to elucidate and generate data from different perspectives (See Table 1).

**Table 1:** Participants in data collection

Haematologist	Gender	Educational status/ Hematological experience	Field Study 1^a^	Field Study 2^b^	In-depth interviews^c^
H1	Male	Medical assistant > 5 years	X		
H2	Male	Medical assistant > 5 years	X		
H3	Female	Medical assistant > 10 years	X		
H4	Male	Senior registrar	X		
H5	Male	Medical assistant > 15 years	X		
H6	Male	Medical assistant > 10 years	X	X	
H7	Male	Medical assistant < 5 years	X		
H8	Male	Medical assistant > 10 years		X	X
H9	Female	Medical assistant		X	
H10	Male	Medical assistant > 10 years		X	
H11	Female	Senior registrar		X	X
H12	Female	Senior registrar		X	
H13	Male	Senior registrar		X	
H14	Male	Medical assistant < 5 years			X

Data was conducted in the phases ^a^15/03/17–16/08/17; ^b^07/03/18–04/05/18; ^c^12/11/18–18/12/18

One month before the intervention study began, haematologists at the department were offered one-hour plenum sessions regarding the purposes and design of the multimethod project. These sessions were held by the researcher responsible for the intervention study, who also was a medical doctor. Sessions included information on how to identify and interpret the PROMs provided in the electronic medical record system. No prescription was provided on how to include PROMs or how to intervene using the information provided in the PROMs, and no clinician alerts were programmed into the medical record system due to the outcomes under examination in the study. Instead, PROMs were intended to be included as relevant to the individual haematologists’ assessment of patient conditions and therefore a part of the department’s existing guidelines for rehabilitation and supportive care for patients diagnosed with haematological cancer [30, 31]. The plenum sessions were repeated after 6 months. Meanwhile, newly employed haematologists received the same in-person informational sessions with the responsible researcher. The information provided at the plenum sessions was also incorporated in a newsletter and distributed via e-mail to all haematologists.

When haematologists consulted with a patient who had completed PROMs, the researcher requested permission to observe the consultation and to conduct a brief interview with the haematologist afterward. All of the haematologists asked consented to the consultation observation; one refused to be interviewed afterward, reporting lack of support in the use of PROMs. All 13 consenting participants were invited to the final in-depth interviews, as was one haematologist who had not previously participated: this last individual was invited strategically as he was engaged with use of PROMs. Two invited haematologists consented to the final in-depth interview: one experienced haematologist and one younger haematologist. In total, 14 participant observations, 13 individual interviews, and three in-depth interviews were conducted with haematologists (one individual was observed and interviewed twice as he was filling in for a colleague). The patients who were present during the observed consultations had already granted consent for these sessions to be observed, as the patients had consented to the multimethod intervention study [29].

The study was conducted at a large outpatient haematology clinic located at a Danish university hospital. The introduction and implementation of PROMs at the hospital was conducted simultaneously with a large restructuring of the hospital’s information technology, including launching EPIC® [32], a multipurpose electronic medical record system.

### Data collection

**Fig. 1:**
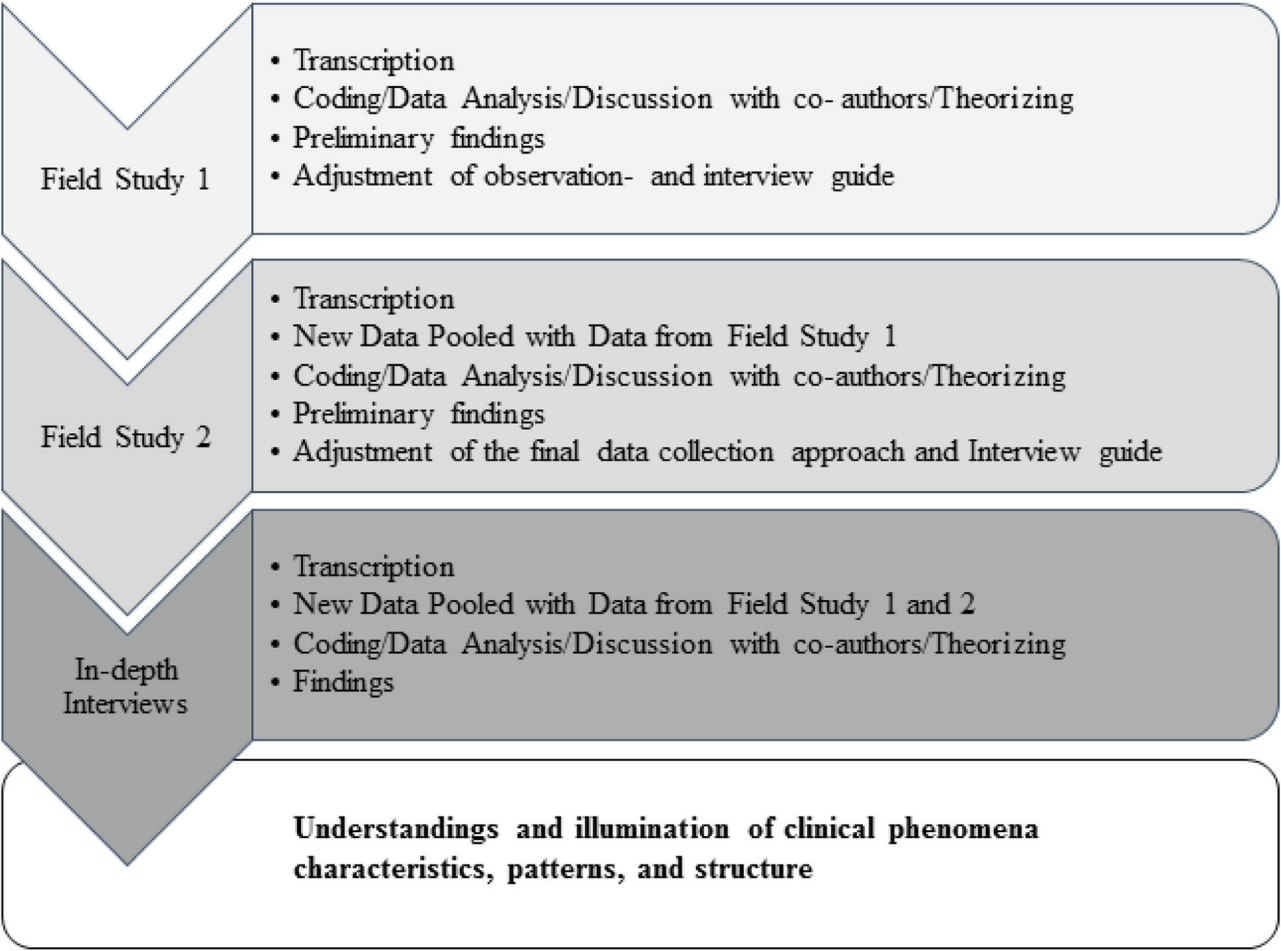
Illustration of the Analytical Processes

Data was conducted from March 2017 to December 2018, consisting of two rounds of participant observations alternating with analysis, and finally the in-depth interviews (see Fig. 1 and Table 1). During the two rounds of participant observations, no haematologists were observed to actively mention or incorporate PROMs, indicating the need for another data source to inform the research. The intention of the participant observations were to observe the haematologists’ attitudes to and use of PROMs in practice. It turned out that the haematologists did not use or refer to the PROMs, which could not have been explored without the observations. Therefore the in-depth interviews were planned, after which the total dataset was judged sufficient to provide answers on the research aim. ID does not seek data saturation, as it is impossible to achieve given human variation and diversity on a topic [33–35]. Instead, the key to quality within an ID study is the internal logic of purpose, process, and context that align into a coherent and convincing account which becomes sufficiently well developed to warrant reporting [24].

Participant observations were carried out by the first author, who was a nurse acquainted with many of the haematologists from a past 4-year tenure as a nurse in the outpatient clinic. Participant observations took place during haematologists’ consultations with patients in the clinic [36]. During the consultation, participant observations followed an observation guide [37]. Field notes were taken during observations and supplied to the researcher immediately after the session [38]. The subsequent interviews were short and focused, as they were conducted in between the haematologists’ consultations appointments, and aimed to explore haematologists’ experiences and reflections related to PROMs [39]. A semi-structured interview guide provided guidance, including descriptive, structural, and contrast questions [40]. The observation and interview guide was developed according to the guidelines of the Consolidated criteria for Reporting Qualitative Research (COREQ) checklist [41] by the first author and co-authors, focusing on investigating the haematologists’ experiences, motives and broader reflections on PROMs. Participant observations, interviews, and in-depth interviews were audio recorded, manually transcribed, and organized into electronic files in NVivo PRO™ [42]. In the presentation of findings, ‘H’ followed by an individual number refers to a specific haematologist (see Table 1). F1 refers to data from Field Study 1, F2 refers to data from Field Study 2, and INT refers to data from the in-depth interviews.

### Data analysis

Data analysis was performed inductively, concurrently with data collection, as a creative and abductive process (see Fig. 1) [24, 43]. Observations and field notes informed the analysis to explain what occurred in clinical practice, the focus (if it was not the PROMs), and contextual information on the clinical environment as a specific setting [23, 44]. The analytical process resulted in a critical appraisal of arguments, discussions, and theorizing of perspectives, resulting in the conceptualization of causality and the revealing of credible and meaningful findings. The analytical process was initially performed in NVivo PRO™ [42]. Additionally, in the final phases of analysis, data was printed on paper to provide a more creative and visualized exploration without locking data into predefined boxes. Moving quotations around in paper form helped to reveal new insights regarding meanings and relationships in the data. The analysis was performed in Danish to avoid language barrier limitations, and quotes translated into English for the presentation of findings.

## Results

The haematologists included were characterised as having distinct, rather antagonistic, and dichotomous views on PROMs. Overall, haematologists resistant to the application of PROMs were characterized as experienced haematologists, mainly males. Haematologists supporting the application of PROMs were younger, both male and female haematologists engaged in medical specialization training, with a mindset predisposed towards practice development. These findings emerged during observations and interviews. No haematologists were observed to directly elaborate on PROMs during observed consultations. Across all observations, primary focus was on the haematological agenda, such as blood test results and medication. The haematologists, who during the interviews declared intentions of using the PROMs, viewed PROM information as additional information or pseudo-data, not mentioned in the patient-doctor interaction. Two overall themes emerged from the analysis, discussed below.

### Theme 1. Against PROMs

Through the analytic process, the first overall theme “Against PROMs” was explored and separated into four subthemes: 1) PROMs address information irrelevant to haematologists but relevant to patients’ general practitioners (GPs), 2) Use of PROMs is time-consuming, 3) PROMs are unnecessary and 4) PROMs are difficult to use. These findings represent an assembled interpretation of haematologists’ experiences with PROMs that were not supportive of their clinical practice.

#### PROMs address information irrelevant to haematologists but relevant to general practitioners

One of the most prevalent observations by haematologists was that PROMs provide patient information irrelevant to haematology. The haematologists interpreted some information as relevant to patients’ GPs. Consequently, the haematologists may have indicated to patients that the department was not interested in patients’ overall health; a discussion of such might have monopolized the brief time allotted to consult on patients’ hematological diseases.

These PROM completions form a serious range of problems. As haematologists, we are nerds in our field … We must be careful when opening up issues like constipation, [because] then we open up issues that we should not be involved with. If I start up solving issues which the patient is already handling with his GP, it will do more harm than good. Normally the patients know where to address their problems … If we ask all these questions with PROMs, patients might think that I can help them solve all sorts of things. But in reality the relevance for me is to control patients’ EPO treatment, which patients’ GPs cannot. With PROMs we end up talking about anything other than what we should … I guess that is the question – is constipation relevant to haematologists or is that an issue for the GP …? (H1, F1).

This quotation raises several issues. First, it is an ethical problem if PROMs identify issues haematologists cannot respond to. The consequences of a PROM could thus lead to more harm than good. Second, there is a lack of recognition on the part of the system of patient knowledge of where to discuss specific health problems, and lack of respect of the general practitioners’ domain. Finally, this haematologist assumed a single-profession perspective, not considering PROMs as a multidisciplinary tool to be used within the department.

#### Use of PROMs is time-consuming

Haematologists were asked about the presentation of PROM data in the electronic medical record system. Some identified the layout as problematic because too much data was reported in light of the time available.

I look at the PROM notes quite briefly. The first time I saw the PROM notes I studied the data more in detail. But the notes are so comprehensive that it takes far too long a time to interpret the data compared to the work flow we have. In the meantime, I imagine – maybe I have high thoughts of myself – that I can easily see if there is a problem relevant to me … The notes are standardized with a large volume but with less substance … I believe for some patients this is going to increase the amount of communication, which is unwanted to me, if I may say so … (H6, F2).

#### PROMs are unnecessary

During the interviews, haematologists related their experiences with PROMs. Most haematologists simply reported not using PROMs as the data was not useful for consultations. A few had very strong feelings against the introduction of PROMs, one explaining that PROMs limited his freedom:

I have to say, as a general rule I don’t use the PROMs. I don’t. I cannot see the intentions behind it. I meet patients that I have known for years throughout my clinical practice, and I ask them how they are doing, I ask about their symptoms. Patients can tell what’s on their mind. I don’t need to send patients a questionnaire to clarify these things, I don’t. I think that the patients are relatively uncomplicated, so why introduce a questionnaire between the patient and me? That does not make sense to me. Clinicians, both nurses and haematologists, have the ability to recognize personalities, and we talk to our patients in relation to that ability as we use our skills … I wish that we could get rid of these PROMs and focus on our work. (H8, INT).

This experienced haematologist argued that the art of patient-centred care centers rather on the art of conversation, not relying on PROMs, and that patient consultations should focus on knowing the human, not data.

#### PROMs are difficult to use

After the participant observations, the haematologists were asked about the usefulness of PROMS. A number of haematologists expressed simply not knowing what to do with the data. These haematologists were not observed to elaborate on the PROMs in any way during the participant observations.

I have to say that I have not engaged with the PROM data. The note is quite long and it seems rather impossible to get an overall impression of the content. Concretely I don’t know what to do about it. (H13, F2).

I have previously worked with PROMs in clinical trials. But I have not tried to use PROMs on an individual level before, and I think it is difficult to take action on the information that I get. (H9, F2).

These quotations could be interpreted in several ways: not only are PROMs difficult to use, but there may also be a lack of training on PROMs, or an expression of uncertainty about PROM measurement validity.

### Theme 2. Supportive of PROMs

The second overall theme “Supportive of PROMs” arose as haematologists expressed that PROMs had or could have a supportive function in their clinical practice. The theme consisted of three subthemes: 1) A better impression of patients’ conditions*,* 2) PROMs can increase practice efficiency, and 3) Patients’ experiences are important*.*

#### A better impression of patients’ conditions

The theme ‘A better impression of patients’ conditions’ was identified as haematologists reflected on the potential of PROMs and how the data could provide new information before patient visits. For example, patients’ blood test data were used during haematologists’ preparation as a pre-assessment of patients’ conditions.

The PROM data provides me with an impression of the patient’s condition before I see them here … then I know how the patient is doing. Normally, we don’t know before the patient is coming and sometimes it is a disaster that is coming through my door. Of course, sometimes I can see it from the blood test as well, that this patient might not be doing well … But sometimes the blood tests are normal, but the patients claim to feel awful. Then you have to identify the problem … and sometimes the problem is a family-related issue, which has nothing to do with this. Such cases takes a long time … But when I have these data in advance, I have a better impression if there is a problem, so I am ready … In that way PROMs are quite positive. (H11, INT).

This haematologist expressed a high level of confidence in the result reported, not questioning if the PROMs measured the patient’s situation accurately or how the data were presented [16, 17, 45].

#### PROMs can increase practice efficiency

Few haematologists who mostly supported the use of PROMs reflected on the Danish healthcare authorities’ decision that PROMs are to be implemented in the treatment of all cancer patients.

A part of this game or project with PROMs is how we can ensure that the patient is doing well, and, being fair, which patients can refrain from consultations. These patients are diagnosed with low-grade malignancies. So which patients could we ask these PROM questions, and then they don’t have to visit the hospital? With two of my patients I have thought that it was rather ridiculous that they had to show up, as the blood test were fine, everything was fine, and the patients were doing really well … Some patients in this region travel from far away to get here for no reason, so I believe that it would make sense to reverse these consultations supported by PROMs. (H4, F1).

This haematologist related the suggestion to her own practice and how this approach could potentially be supportive for patients and the department.

#### Patients’ experiences are important

Aside from the theme ‘PROMs address information irrelevant to haematologists and relevant to general practitioners’, the issue that most haematologists, both supportive of and resistant to the introduction of PROMs, addressed during the interviews was the part of the PROMs reporting on patient satisfaction. Haematologists reported that patients’ assessment of the treatment and care provided assisted in evaluating their own practice.

The only part of PROMs that I use is the part about patients’ satisfaction … If I had patients who were dissatisfied with the communication from me or my colleagues, then I guess it would be obvious through PROMs … So patient satisfaction is bothering me and an issue that I am interested in when reading the PROM data … And if the patient is dissatisfied then we can discuss that … But I have never experienced that yet… (H6, F1).

## Discussion

In the present study, the haematologists were characterized with two distinct attitudes towards PROMs: first, resistant to the use of PROMs, mainly expressed by haematologists who were critical of data-driven decisions and applying an instrument to the art of conversation and patient-centred care. Second, younger haematologists mainly experienced PROMs as adding new and relevant information which could potentially lead to new types of consultations. However, these younger haematologists assumed that the PROMs were previously validated and utilised accurate measurement properties.

The outcome “patient satisfaction” was referred to as the most important outcome for the haematologists no matter their gender, experience and attitudes towards PROMs. The interest in satisfaction might be interpreted as haematologists fearing patient dissatisfaction and its consequences; this rendered PROM outcomes merely interesting as an evaluation of haematologists, rather than one relevant to patient care. Other studies have found that physicians fear receiving complaints as they were interpreted as ‘mistakes’ made within a highly competitive profession, something with largely psychological consequences for the individual haematologist and their future practice [46–48]. This fear of complaints exemplifies how the underpinning legislation and systems rule and influence haematologists’ practice.

**Fig. 2:**
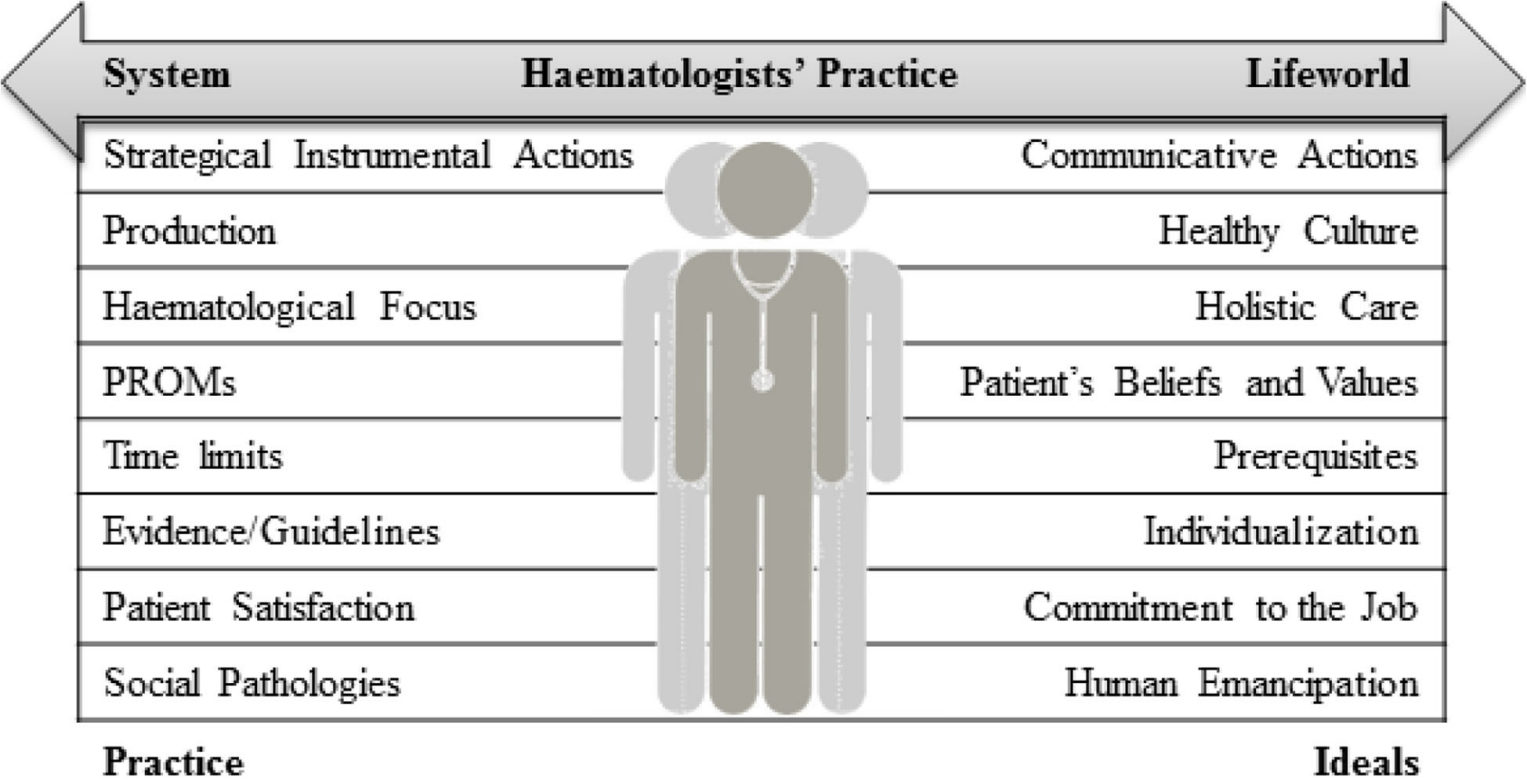
Characteristic Dichotomies in Hematologists’ Practice Inspired by Habermas and McCormack

Following a critical theoretical perspective and using ID to generate knowledge for practice, when interpreting these findings it is essential to contextualize the healthcare system haematologists operate in and to identify inherent control mechanisms ruling haematology practice [49, 50]. In Habermas’ framework, haematologists’ practices exist in tension between the system and the lifeworld (see Fig. 2). Haematologists themselves represent the system, as they are a profession controlled by legislation, organizational structures, guidelines, specialization, and, pragmatically, a daily schedule in the outpatient clinic with strict time limits to manage patient flow. Furthermore, the pre-defined and standardized context allows a very brief time for core duties such as updating patient condition status and treatment planning during appointments [2, 51, 52]. Consultations are thus dominated by the haematological biomedical agenda, one set and governed by the system, leaving haematologists with no choice regarding priorities during consultations. Time for communicative actions and exploring the lifeworld was limited to small talk between a few haematologists and their patients, such being the circumstances allowed by the system [49, 50].

PROMS were implemented in this department simultaneously with the implementation of a new mandatory electronic system. This may explain why some haematologists did not use the PROMs, instead following the system’s logic and prioritizing mandatory tasks, especially when the haematologist did not find PROMs useful. Some even regarded the introduction of PROMs as a top-down decision disrupting the patient-doctor relationship. A previous study on training clinicians to use PROMs found that a key issue limiting implementation was clinicians’ lack of knowledge on how to apply PROMs during clinical encounters [53]. Another implementation study pointed out the importance of stakeholder buy-in as a prerequisite to implementation, as individuals can play a critical role in helping to adopt usage of PROMs [54]. A defined, procedural framework might help clinicians value the information obtained from PROMs and understand that they can facilitate shared decision-making and person-centered care [55–57]. Comparing our study to this literature, the introduction of PROMs without a focus on stakeholder buy-in or procedural usage recommendations may have been naïve, which is a limitation to our findings.

Looking at the themes identified among those supportive of the use of PROMs, some are compatible with some existing knowledge, such as that PROMs potentially provide a better impression of patient conditions and can increase practice efficiency, and that patient satisfaction is important [2, 4, 15]. However, our findings were characterized merely as potential supportive features expressed by haematologists: concrete application of PROMs data was absent, rendering these findings uncertain.

A recent study aimed to determine if PROM data was a valuable tool to assess health-related quality of life (HRQoL) among patients diagnosed with multiple myeloma [58]. The study identified a range of methodological challenges: in order to make HRQoL meaningful, patients might adapt to changes in HRQoL over time, rendering the measurements unreliable, and also patients were liable to not complete PROMs as their disease progressed. These findings could be interpreted as lack of content validity, something that was also identified in the present study when analysing patients’ experiences with PROMs [29]. Another study from neurology investigated PROM-based outpatient follow-ups and concluded that use of PROMs could influence the patient-doctor interaction, increasing patient involvement; this was mainly related to pharmacologic treatment, but the study also found ambivalence among clinicians, as PROMs could both improve and impair the quality of follow-ups [59]. Comparing previous research with our results, a discussion about the value of PROM data is needed, as it appears that introduction of PROMs in clinical practice is linked with some disadvantages. Overall, the approach of using PROMs to identify patients’ individual needs and provide patient-centred care must be questioned, as the quantification of individual experiences through standardised questionnaires is linked with lack of content validity and inadequate psychometric measurement properties [16, 17, 45]. Also, the PROM instrument applied should be considered; in our study the instrument was associated with low content validity and lack of item consistency [45]. Patients did not find questions relevant to their disease and a large number of patients requested free-text entry boxes, indicating a lack of coverage, or that none of the responses were aligned with their situation, leading to potentially invalid responses [29]. The haematologists also did not find the outcomes relevant to their practice and did not understand the PROMs’ scope. Finally, one should be critical towards applying a quality of life questionnaire, as quality of life is not easily quantified through predefined items but rather more as an individual judgment of the value of life circumstances [60, 61]. This point was supported by haematologists in our study as they did not evaluate PROMs as adding precise information about the individual patients, a finding consistent with the imprecision of PROMs when used at the level of individuals and not a group of people [17]. Our findings elucidate how PROMs represent the system, adding another layer of bureaucracy and limiting haematologists’ possibilities for communicative actions, including supporting a patient-centred care culture [62]. This was contrary to the system’s rhetorical insistence on PROMs as meaningful data work [13]; PROMs became meaningless busywork with low legitimacy in some of the haematologists’ clinical practice [11, 21].

## Conclusions

The introduction of PROMs in this hematological outpatient clinic did not lead to incorporation of patient information or clinician elaboration on PROMs in the patient-doctor relationship, leaving uncertainties about the potential of PROMs and specifically about the instruments applied. The haematologists were characterized by antagonistic, dichotomous attitudes toward PROMs, either supportive of or resistant to their use. Supportive haematologists were mainly younger, while resistant haematologists were more clinically experienced and critical of the subject the PROMs encompassed. Haematologists experienced patient satisfaction as the most important outcome, while the remaining information from the PROMs was mostly irrelevant to haematologists who did not have the time or ability to address additional symptoms.

### Practice implications

First, if PROMs are to actuate their future potential within clinical haematology practice, clinicians and other stakeholders should be involved and engaged throughout the preparation stages, to improve adoption and to encourage support and usage. Second, future PROM instruments should be psychometrically tested, adjusted, and validated within the specific patient group to ensure content reliability and relevance. Finally, one should be aware of the pitfalls associated with adapting PROMs to improve an individualized approach in clinical practice. Other approaches, such as the patient-centred care framework [63], might be more appropriate when aiming to improve individualized care.

## Supplementary information

**Additional file 1:** Overview of the multimethod study.

**Additional file 2:** Additional information on the PROMs implemented.

## Data Availability

The datasets generated and/or analysed during the current study are not publicly available but are available from the corresponding author on reasonable request.

## Abbreviations

EORTC QLQ-C30: European Organization for Research and Treatment of Cancer Quality of Life Questionnaire Version 3.0
GPs: General practitioners
HRQoL: Health-related quality of life
ID: Interpretive Description
OEQ: Outcomes and Experiences Questionnaire
PROM: Patient Reported Outcome Measures
