# Supplementary material for: Haematologists’ experiences implementing patient reported outcome measures (PROMs) in an outpatient clinic: a qualitative study for applied practice
Source: J Patient Rep Outcomes. 2019 Dec 28;3:74. doi: 10.1186/s41687-019-0166-6 (PMC6935381; doi:10.1186/s41687-019-0166-6)

***Supplementary Material 2.*** *Table 1 adapted as original from the publication:*

Thestrup Hansen, S., Kjerholt, M., Friis Christensen, S., Brodersen, J., & Holge-Hazelton, B. (2019). “I Am Sure That They Use My PROM Data for Something Important.” A Qualitative Study About Patients’ Experiences From a Hematologic Outpatient Clinic. *Cancer Nursing*. <https://doi.org/10.1097/NCC.0000000000000738>


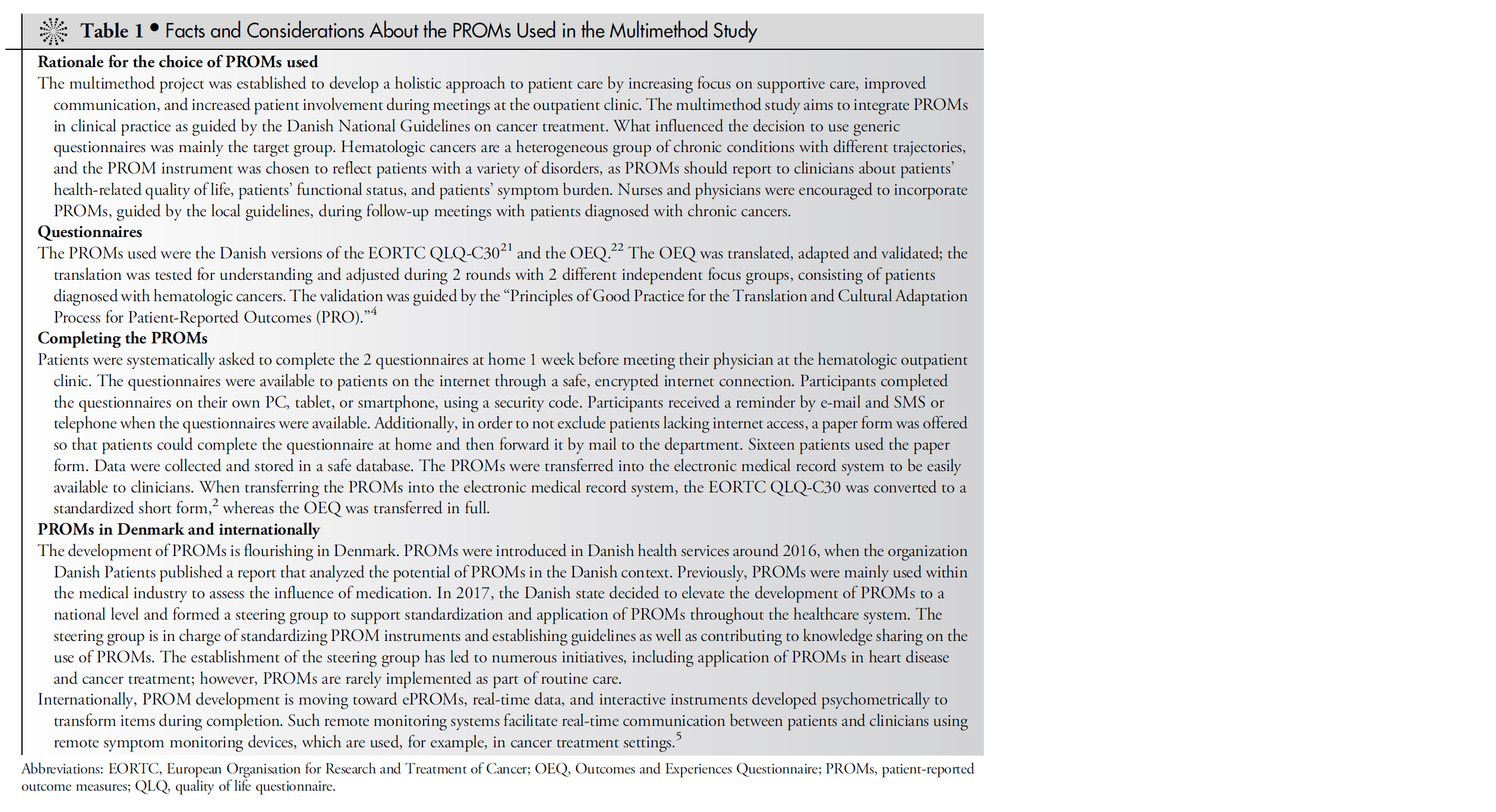

Supplement: Supplementary file 2 — Additional file 2: Additional information on the PROMs implemented. [file 41687_2019_166_MOESM2_ESM.docx]
